# Combined long-term enriched environment and caffeine supplementation improve memory function in C57Bl6 mice

**DOI:** 10.1007/s00406-022-01431-7

**Published:** 2022-06-09

**Authors:** Martina Stazi, Silvia Zampar, Madeleine Nadolny, Luca Büschgens, Thomas Meyer, Oliver Wirths

**Affiliations:** 1 Department of Psychiatry and Psychotherapy, University Medical Center (UMG), Georg-August-University, Von-Siebold-Str. 5, 37075 Göttingen, Germany; 2 Department of Psychosomatic Medicine and Psychotherapy, University Medical Center (UMG), Georg-August-University, Göttingen, Germany

**Keywords:** Physical activity, Caffeine, Enriched environment, Behavior, Neurogenesis, Wildtype mice

## Abstract

Regular physical activity has been associated with healthy brain aging, reflected by beneficial effects on cognition and learning and memory. Nutritional supplements such as caffeine have been shown to act as cognitive enhancers and may possess neuroprotective properties. Interestingly, caffeine also improves athletic capabilities and is widely used by athletes because of its performance-enhancing effect, while information on potential additive beneficial effects of physical activity and caffeine on cognitive performance is scarce. In the present study, the effects of caffeine supplementation in combination with prolonged physical and cognitive stimulation in the form of the enriched environment (EE) housing for a duration of 4 months were analyzed. We demonstrate that caffeine supplementation together with prolonged environmental enrichment led to enhanced memory function, resulting in improved recognition and spatial working memory in behavioral paradigms such as the novel object recognition task or the Morris water maze in C57Bl6 wild-type mice. Mice housed under EE conditions showed increased gene expression levels of brain-derived neurotrophic factor (BDNF) in the hippocampus. The present findings underscore the potential impact of continuous physical activity in the prevention of age-related cognitive decline and may offer new options for combinatorial approaches.

**Supplementary Information:**

The online version contains supplementary material available at 10.1007/s00406-022-01431-7.

## Introduction

Several nutritional supplements are known to have ergogenic properties on endurance performance [10], with caffeine being one of the most extensively studied. Caffeine, an antagonist of the adenosine receptor [14, 46], is a widely consumed performance-enhancing dietary supplement utilized by athletes showing positive effects on parameters such as mean running speed or total distance [15, 16]. The first known study regarding the performance-enhancing effects of caffeine dates back to 1907 [58], and since this study, the interest in caffeine consumption with regard to sport performance has grown continuously. Nowadays caffeine is a common substance in the diets of most athletes and regarded as a powerful ergogenic aid in training and competition [22]. Caffeine can be ingested from natural sources (e.g. coffee and tea etc.) or can be artificially synthesized and added to food and drinks (such as energy drinks) [15], however, the ingestion of coffee appears to be ineffective compared to pure caffeine [22]. It has been shown that caffeine can improve sport performance if ingested at low-moderate dosages (3–6 mg/kg), while higher dosages (≥ 9 mg/kg) do not result in further enhancement [21]. Several meta-analyses examining the impacts of caffeine ingestion on physical activity have been done, and all of them point out to the fact that caffeine can reliably enhance exercise performance [23, 62].

With regard to cognitive functions, caffeine’s properties have been analyzed in both human and animal studies. Longitudinal reports suggest that chronic coffee/caffeine consumption might exert neuroprotective properties and helps to prevent cognitive impairment in aging humans [57, 65]. Moreover, a link between habitual caffeine intake and Alzheimer’s disease (AD) diagnosis has been described in an epidemiological report. In this case–control study, control patients presented with significantly increased caffeine intake during a 20 years period compared to age-matched AD-affected individuals, resulting in a significantly lower risk for AD, independently of other confounding variables [47]. Moreover, preclinical studies employing AD transgenic mice have shown that long-term oral caffeine ingestion results in sustained reductions in plasma β-amyloid (Aβ) levels, as well as decreased soluble and deposited Aβ in brain regions such as hippocampus and cortex [4, 8].

The beneficial effects of exercise on the aging human brain have been repeatedly reported and it has been shown that both acute and moderate-intensity exercise (and caffeine) are able to significantly improve working memory [53]. Weuve and colleagues have demonstrated that long-term physical activity results in less cognitive decline and enhanced cognitive performance in older women [69] and a recent Finnish twin study showed that long-term vigorous physical activity in adulthood is significantly linked to reduced dementia risk in later life [35]. A multitude of studies employing animal models have been carried out in recent years to understand the underlying mechanisms of physical activity and beneficial effects on cognition. Housing rodents in an enriched environment condition (EE) [55], to mimic an active lifestyle in terms of cognition and physical exercise [39], has become the predominating paradigm to investigate this relationship in preclinical settings.

To the best of the author’s knowledge, no studies have examined the possible effects of prolonged caffeine treatment in combination with EE and physical exercise on healthy mice. The purpose of this study was to investigate the potential synergistic effects of prolonged caffeine ingestion and housing in EE conditions on behaviour, neurogenesis as well as hippocampal neuron number of healthy C57BL/6J animals.

## Material and methods

### Animals and treatments

**Fig. 1:**
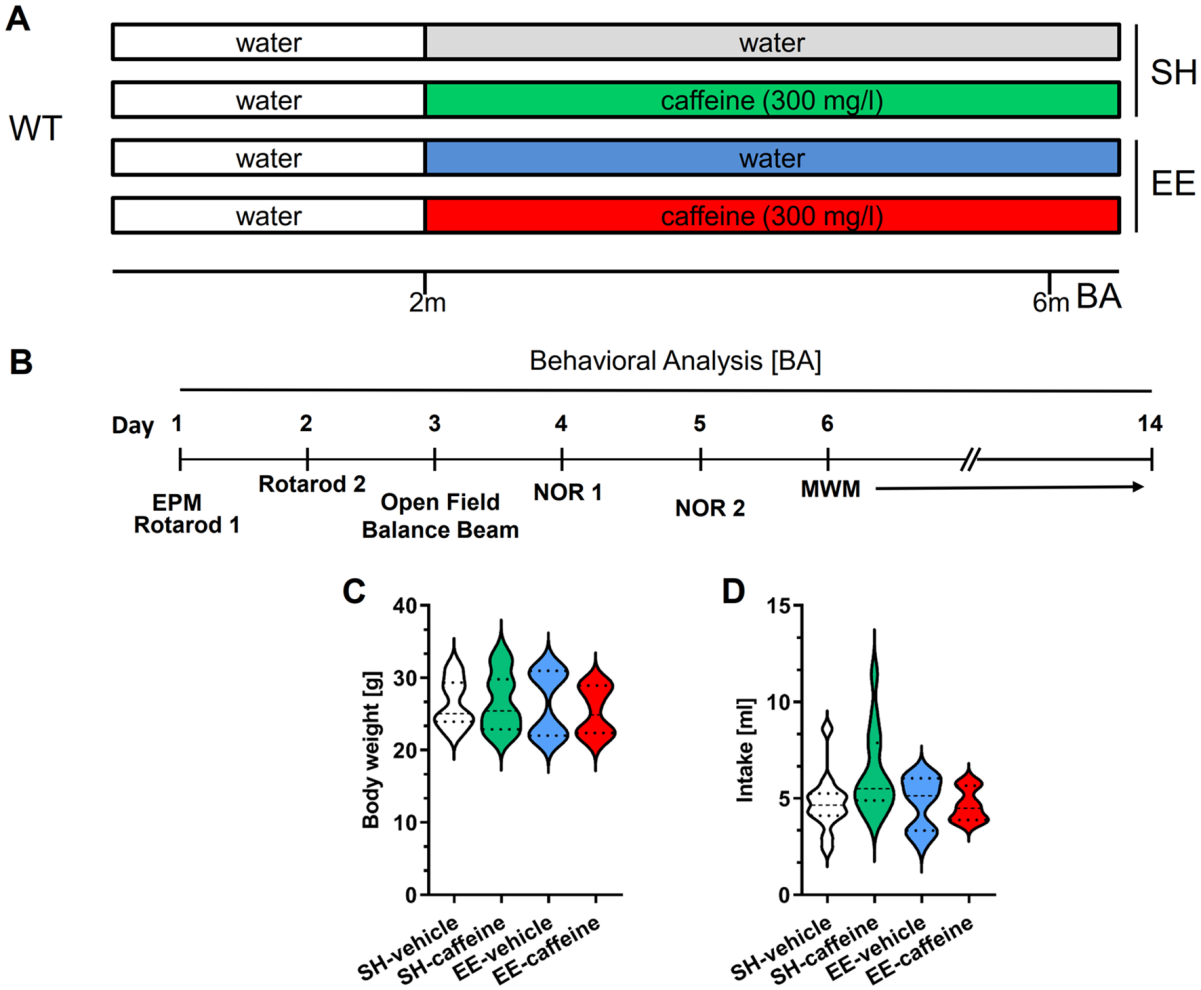
Experimental design and housing conditions. At 2 months of age, WT mice were either assigned to standard (SH) or enriched environment (EE) housing and received tap water or caffeine-supplemented drinking water (**A**). At six months of age, mice were analysed with a battery of motor as well as learning and memory tasks with ongoing treatment or EE housing (**B**). Daily water consumption and body weight assessment during the behavioural test analysis (BA). Caffeine treatment and EE housing did not affect the bodyweight of the WT animals (**C**) and liquid intake was similar between all the groups (**D**). One-way ANOVA followed by Bonferroni’s multiple comparisons test. All data were given as mean ± SD

In this study, an equal number of female and male C57BL/6J mice (Jackson Laboratories, Bar Harbor, ME, USA) were used. At 2 months of age, wild-type (WT) mice from the strain C57BL/6J were randomly assigned to either standard housing (SH) or EE conditions until the age of 6 months [64]. While mice in SH conditions were kept in standard laboratory cages (33 cm × 18 cm × 14 cm), larger rat cages (55 cm × 34 cm × 20 cm) equipped with running tunnels, plastic and metal wheels, nesting material, houses and toys were used in case of EE housing, which were cleaned and rearranged weekly to increase the sense of novelty, as done in previous studies [33, 64]. In further groups of animals housed in SH and EE conditions, chronic oral caffeine treatment was initiated at 2 months of age (Fig. 1A). Caffeine (Sigma-Aldrich) was administered to the animals via drinking water at a dose of 300 mg/L, which has been shown to correspond to ~ 5 cups of coffee per day in humans [3, 4] and administration was maintained during behavioural testing. Control groups received tap drinking water (vehicle) and water consumption and body weight were measured daily throughout the behavioural test phase to assess comparable drug intake. Mice were housed in groups of 4–5 in all conditions to ensure social interactions; food and water were provided ad libitum*.* Data from vehicle- and caffeine-treated SH groups have been partially published in a previous study [63], where they served as control groups within a larger set of experiments to minimize experimental animal numbers. All animals were handled according to the German guidelines for animal care and all experiments have been approved by the local animal care and use committee (Landesamt für Verbraucherschutz und Lebensmittelsicherheit (LAVES), Lower Saxony, Approval number 17/2701).

### Behavioural testing

To assess the potential beneficial effects of prolonged caffeine treatment and EE with regard to learning and motor behaviour, mice were tested at 6 months of age at the end of the treatment period in a set of anxiety, motor and memory tests (*n* = 10–14 per group; Fig. 1B). Animals were kept on a 12 h/12 h inverted dark/light cycle (light phase between 8 p.m. and 8 a.m.) and were sacrificed immediately after the last day of testing. All behaviour experiments were carried out during the dark phase.

#### Elevated plus maze

The Elevated Plus Maze (EPM) was used to evaluate exploratory behaviour and anxiety levels [36]. Briefly, the EPM consisted of an apparatus with a cross shape (four arms of 15 cm length and 5 cm width), lifted 75 cm above a padded surface. While two oppositely positioned arms contained lateral walls (“closed arms”), the other pair of arms was open. Mice were placed in the centre of the EPM facing one of the open arms and were allowed to explore the apparatus freely for 5 min. Several parameters such as distance travelled, speed, arms entries and percentage of time spent in each arm were recorded and calculated using a video tracking system (ANY-Maze, Stoelting Europe, Dublin). Anxiety-like behaviour was assessed using time spent in the open arms, with longer periods corresponding to reduced anxiety levels [37]. The EPM was cleaned between animals with 70% ethanol to eliminate odour cues.

#### Accelerating rotarod

Motor learning, motor performance and balance abilities were analyzed using the accelerating rotarod test [60] (RotaRod, TSE Systems GmbH, Bad Homburg, Germany). The test was performed on 2 consecutive days with 4 trials per day and at least 15 min inter-trial intervals. Each mouse was individually placed on the rod, which accelerates from 4 to 40 revolutions per minute (rpm) over a maximal trial time of 300 s. The time spent on the rod was recorded as an indicator of motor performance [latency to fall (s)] and trials were terminated when animals fell off or the maximum time was reached. The apparatus was cleaned between trials with 70% ethanol to avoid odour cues.

#### Open field and novel object recognition

The open field (OF) test was used to investigate locomotor activity, exploratory behaviour and anxiety levels. During the OF test, mice were placed in the middle of a square arena (50 × 50 cm) where they could freely explore the area during a single 5 min trial. The total time spent in the central part of the arena, the total distance travelled, as well as the average speed were recorded using video-tracking software (ANY-maze, Stoelting Europe). Twenty-four hours after the OF, the Novel Object Recognition test (NOR) was performed in the same arena, now having two identical objects (training phase). The NOR is a commonly used behavioural assay to test various aspects of learning and memory in rodents, especially recognition memory and novelty preference [2]. Metected among all experimental groups. During tice were allowed to freely explore the objects for 5 min. Twenty-four hours later, one of the 2 objects was replaced with a novel one consistent in height and volume but different in shape and appearance (testing phase). Object exploration was scored whenever the mouse sniffed the objects while looking at them, whereas climbing onto the object was not considered as exploration [44]. Data collection and video analysis were performed in blind to the experimental conditions. The percentage of exploration time for the novel object was calculated as follows:

$$= \frac{{\left( {{\text{time at novel}} \times 100} \right)}}{{{\text{total}} \,{\text{exploration}}\, {\text{time}}}}$$

In addition, observation scores were converted into discrimination indices (DI) to define novel versus familiar object exploration rates:

$$= \frac{{\left( {{\text{time}} \,{\text{at}} \,{\text{novel}} - {\text{time}} \,{\text{at}} \,{\text{familiar}}} \right)}}{{{\text{total}} \,{\text{exploration}} \,{\text{time}}}}$$

In between trials, the arena as well as the objects were cleaned with 70% ethanol to diminish odour cues.

#### Morris water maze

The Morris water maze test (MWM) [54] was used to assess spatial reference memory as previously described [5]. In brief, mice were trained to learn to localize a submerged platform (ø 10 cm) in a circular pool (ø 110 cm). The pool was filled with water made opaque with non-toxic white paint to make the platform invisible and to facilitate the video tracking. First, a “cued training” was carried out on 3 consecutive days (4 trials per day), in which the submerged platform was marked with a triangular flag. Twenty-four hours after the last trial of the cue training, mice performed 5 days of “acquisition training” (4 trials per day). In this training block, proximal cues were added around the pool and the triangular flag was removed from the platform, which remained stationary for each mouse. A “probe trial” was used to assess spatial reference memory 24 h after the last trial of the acquisition training. During this 1 min trial, the platform was removed while proximal and distal cues remained. Since the platform location was kept constant during the acquisition training phase, mice with successful spatial reference memory consolidation should exhibit a target quadrant preference. Between the trials, mice were kept under infra-red light to dry and prevent hypothermia. All trials were recorded using a video tracking software (ANY-maze, Stoelting Europe) and parameters such as escape latency, swimming speed, swimming path quadrant preference, latency to first entry into the platform/target quadrant, time into the platform/target quadrant and entries into the platform/target quadrant were analyzed.

### Tissue collection and preservation

A subset of mice was deeply anesthetized and transcardially perfused using ice-cold phosphate-buffered saline (PBS) or euthanized with CO_2_ asphyxiation and cervical dislocation before brains were carefully dissected. The right hemisphere was post-fixed in 4% formalin solution at 4° for at least 72 h protected from light before embedding in paraffin. The left hemisphere was postfixed in 4% paraformaldehyde (PFA) in PBS for at least 24 h before being transferred to a 30% sucrose solution (in PBS) for cryo-protection. Brain tissue of the remaining mice was deep-frozen on dry ice and stored at -80 °C until further use.

### Quantification of CA1 neuron numbers

Neuronal quantification in the CA1 region of the hippocampus was done on 4 μm sagittal paraffin brain sections (Bregma lateral 1.08–1.32) cut on a rotation microtome (Microm, HM335E, Thermo Fisher Scientific, Germany) and stained with haematoxylin. Neuronal nuclei were determined by their size and peculiar appearance clearly differing from glial cells. Images of the CA1 area of the hippocampus were acquired at ×400 magnification using an Olympus BX-51 microscope equipped with a Moticam pro 282 camera (Motic, Germany). The number of CA1 neurons per section (*n* = 3 per animal, 40 µm intersection distance) was counted using the manual cell counting tool implemented in ImageJ (version 1.52u, NIH) and data were normalized to WT-SH as the reference group. The experimenter was blinded with regard to genotype and treatment throughout all the analysis.

### Analysis of adult neurogenesis

Frozen cryo-protected brain hemispheres were cut into a series of 30 μm thick coronal sections using a cryostat (CM1850 UV, Leica, Germany). A series of every 10th coronal frozen section was processed in a free-floating staining protocol to quantify the number of newborn neurons. First, one brain section series was rehydrated for 10 min with ice-cold PBS and endogenous peroxidase activity was blocked by immersion in 30% H_2_O_2_ in PBS for 30 min. Sections were washed in PBS containing 0.01% Triton X-100 for membrane permeabilization. Unspecific blocking was done for 1 h by incubation in PBS including 10% fetal calf serum (FCS) and 4% milk powder at room temperature (RT). Primary goat antibody against doublecortin (DCX, sc-8066, 1:500 Santa Cruz Biotechnology, RRID:AB_2088494) was diluted in PBS containing 10% FCS and incubated overnight at RT. On the next day, sections were thoroughly washed with PBS incl. Triton X-100 and incubated with a secondary anti-goat biotinylated antibody (DAKO, Glostrup, Denmark). Staining was visualized by means of the ABC method using a Vectastain kit (Vector Laboratories, Burlingame, USA) and DAB as the chromogen. The total number of newborn neurons was counted in the dentate gyrus (DG) using the meander scan option of StereoInvestigator 7 (MicroBrightField, Williston, USA) to quantify all DCX-positive cells in a given section (8–10 sections per animal). The resulting neuron number was multiplied by 10 to obtain the total number of newborn neurons per hemisphere [12]. The quantification of DCX-expressing cells has been demonstrated to allow for an accurate measurement of modulations in the rate of adult neurogenesis [13]. Volume information was obtained by measuring the area on all analyzed sections and taking into account the corresponding actual average section thickness, as well as the intersection interval, by means of Cavalieri’s principle as done in previous studies [20, 32]. The experimenter was blinded with regard to genotype and treatment throughout the entire analysis. To avoid possible bias due to gender-dependent differences in brain size, for the quantification of CA1 neuron numbers, adult neurogenesis and DG volume, only female mice were used (*n* = 5–6 per group).

### Real-time PCR

Real-time PCR analysis was performed as described previously [31]. In brief, deep-frozen hippocampi (*n* = 5–6 per group) were homogenized in 1 ml TriFast reagent (Peqlab) per 100 mg tissue with a glass-Teflon homogenizer (10 strokes, 800 rpm) and RNA was isolated according to the protocol of the manufacturer. Following a DNAseI (Thermo Fisher) digestion step, reverse transcription was carried out with the RevertAid First Strand cDNA Synthesis kit (Thermo Fisher) according to the protocol of the supplier. RT-PCR was performed on a CFX Connect Real-Time cycler (Bio-Rad), employing the iTaq Universal SYBR Green Supermix (Bio-Rad) for amplification. Primer sets were purchased from Eurofins Genomics as intron-spanning primer sets. Expression levels were calculated using the 2^−CT^ method [45], normalized to *β-Actin* and calibrated to the average expression levels of untreated standard-housed animals for each gene [28].

### Statistical analysis

Differences between groups were tested with either one-way or two-way analysis of variance (ANOVA) followed by Bonferroni’s multiple comparison tests. Significance levels were determined as follows: ****p < 0.0001; ***p < 0.001; **p < 0.01; *p < 0.05. All statistics were calculated using GraphPad Prism version 9.3 for Windows (GraphPad Software, San Diego, CA, USA). MWM probe trial results were analyzed with Dirichlet distributions as described previously [48] using the Dirichlet package from Eric Suh (Fitting the parameters of a Dirichlet distribution) available from: https://github.com/ericsuh/dirichlet.

## Results

To monitor general health and comparable drug intake, body weight and average daily water intake were recorded during the behavioural testing period. No significant differences in body weight (Fig. 1C) or water consumption (Fig. 1D) were detected among all groups, irrespective of the treatment and housing condition.

### Caffeine treatment alters anxiety behaviour in C57BL/6J mice

**Fig. 2:**
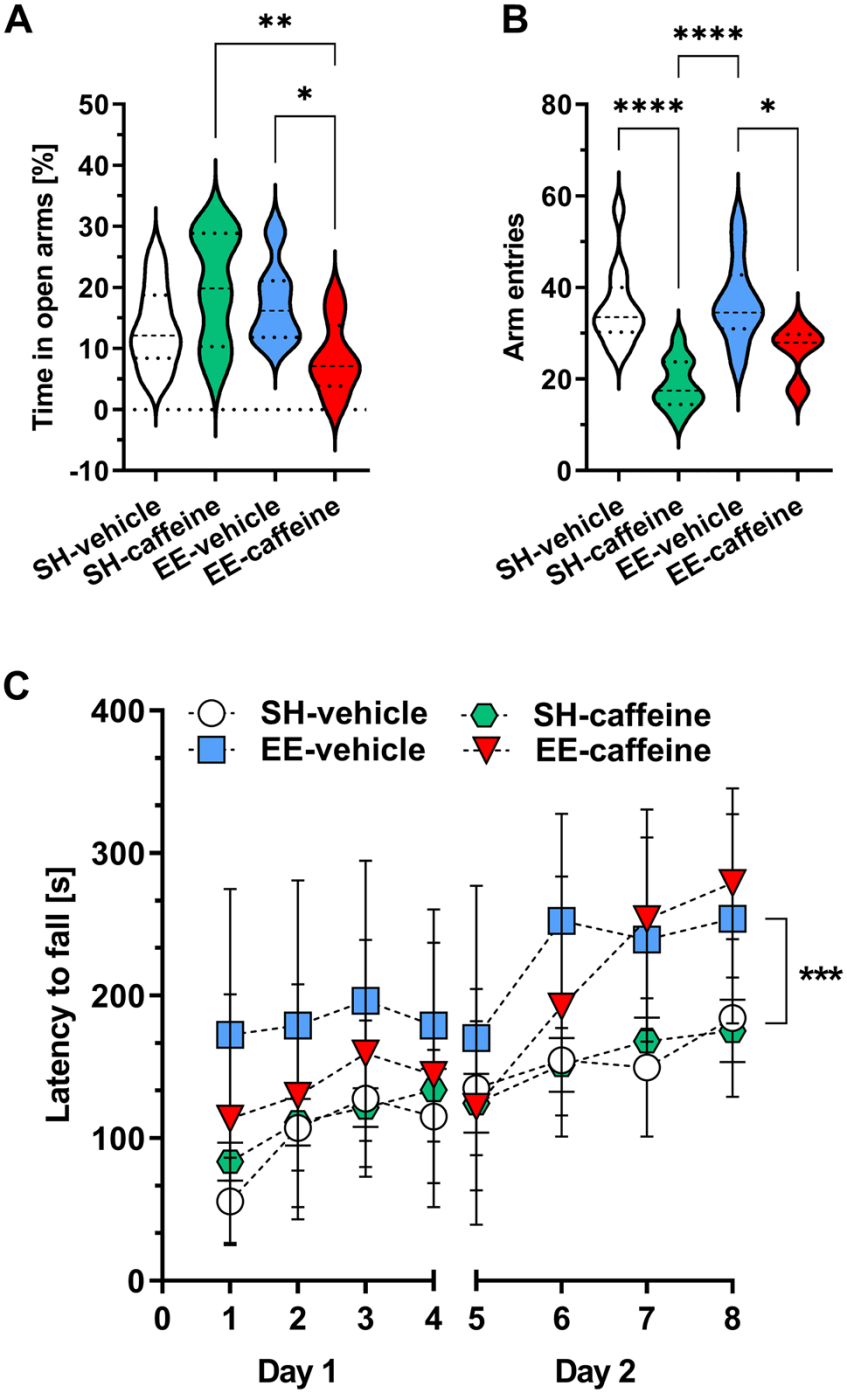
Effects of prolonged caffeine treatment and EE housing on anxiety-related and motor behaviour. **A** Caffeine-treated EE animals displayed an increased anxiety phenotype in comparison to SH mice caffeine and EE mice without caffeine supplementation, as shown by the percentage of time spent in the open arms of the EPM. **B** Caffeine-treated SH and EE mice also showed a significantly reduced number of overall arm entries compared to animals without caffeine intake. **C** Untreated mice housed under EE conditions showed a significantly improved motor performance in the accelerating rotarod task compared to vehicle- and caffeine-treated SH mice. One-way (**A**, **B**) and two-way ANOVA (**C**) followed by Bonferroni’s multiple comparisons test *p < 0.05, ***p < 0.001, ****p < 0.0001. All data were given as mean ± SD

There was no significant difference in the amount of time spent in the open arms of the EPM between vehicle-treated EE-housed animals compared to the WT-SH group. In contrast, caffeine treated mice showed a significant decrease in the time spent in the open arms when they were housed under EE conditions (both *p* < 0.05) (Fig. 2A). Both the SH-caffeine group as well as the EE-caffeine group showed a reduced number of arm entries compared to their respective vehicle-treated counterparts (both *p* < 0.0001 and *p* < 0.05 respectively) (Fig. 2B).

### Improved motor performance in C57BL/6J mice upon enriched environment housing

To investigate whether housing conditions and caffeine treatment have an influence on motor performance in the C57BL/6 J mice, motor function was analyzed using the accelerating rotarod test. All experimental groups improved their ability to stay on the rod during the trials in the course of the two days of testing. While caffeine treatment did not show improved motor performance compared to the respective vehicle conditions, vehicle-treated EE-housed mice performed significantly better than the standard-housed vehicle- or caffeine-treated groups (both *p* < 0.001) (Fig. 2C).

### Combined enriched environment and caffeine treatment improve recognition and working memory

**Fig. 3:**
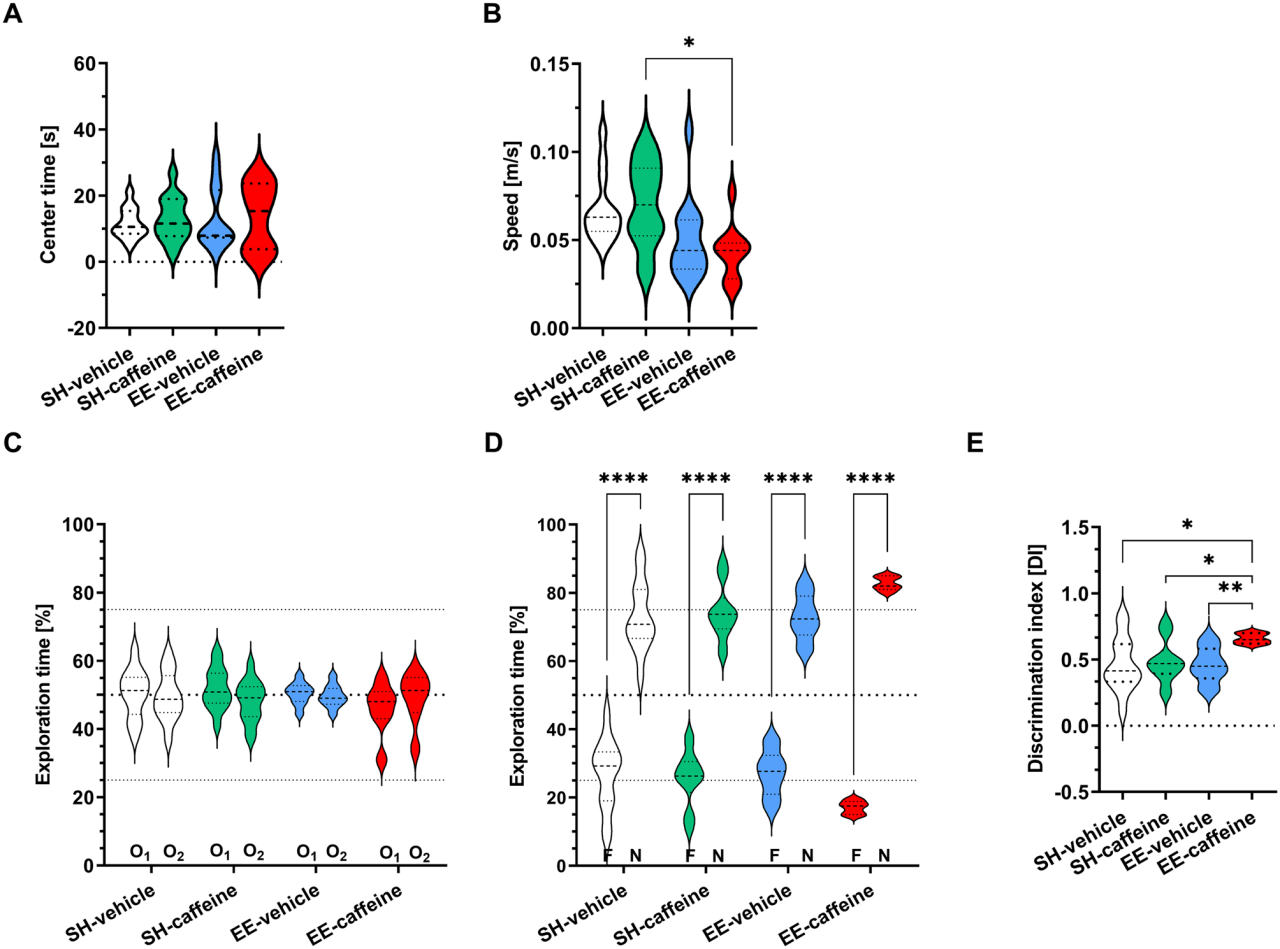
The influence of caffeine treatment and EE housing on recognition memory performance. The novel object recognition (NOR) task was used to test recognition memory. **A** No significant differences were detected in the open field task between untreated and caffeine-treated groups with regard to the time spent in the centre. **B** In caffeine-treated animals, EE housing resulted in a reduced overall speed in the OF preceding the NOR task compared to the SH group. **C** During the training phase on day one, all experimental groups spent an equal amount of time with each of the two similar objects (O1, O2). **D** Twenty-four hours later, during the testing phase, all the groups spent significantly more time with the novel (N) compared to the familiar object (F). **E** Calculation of the discrimination index (DI) revealed that caffeine-treated EE mice performed significantly better compared to the three other groups, indicating a better ability for novelty discrimination. One-way (**A**, **B**, **E**) and two-way ANOVA (**C**, **D**) followed by Bonferroni’s multiple comparisons test. *p < 0.05, **p < 0.01; ****p < 0.0001. All data were given as mean ± SD

The open-field test represents the habituation phase for the novel object recognition (NOR) task, which was used to analyse object recognition memory and novelty preference. No obvious differences among the genotypes were detected in the OF task (Fig. 3A), though caffeine-treated EE mice showed a significantly reduced speed in comparison to caffeine-treated SH mice (Fig. 3B). On the exploration day of the NOR, all groups spent an equal amount of time with the two identical objects (Fig. 3C). When tested for recognition memory 24 h later, all groups spent significantly more time with the novel (N) compared to the familiar object (F), indicating intact object recognition memory (all *p* < 0.0001) (Fig. 3D). A calculation of the discrimination index (DI) showed that EE/caffeine mice present with a significantly higher mean discrimination index as compared to all other groups (*p* < 0.05 and *p* < 0.01 respectively), indicating an additive effect of physical activity and caffeine supplementation on recognition memory (Fig. 3E).

### Improved spatial reference memory upon combined caffeine treatment and EE housing

To analyse whether long-term EE housing in combination with caffeine treatment leads to an amelioration of spatial reference memory, the Morris water maze (MWM) test was performed. The animals in all four groups showed progressively decreased escape latencies over three days of cued training and no difference in swimming speed (Suppl. Fig. 1A, B). In the following acquisition training phase, no difference in spatial learning was detected among all experimental groups. During the entire cued and acquisition training phases, all groups showed comparable escape latencies and speed (Suppl. Fig. 1C, D).

**Fig. 4:**
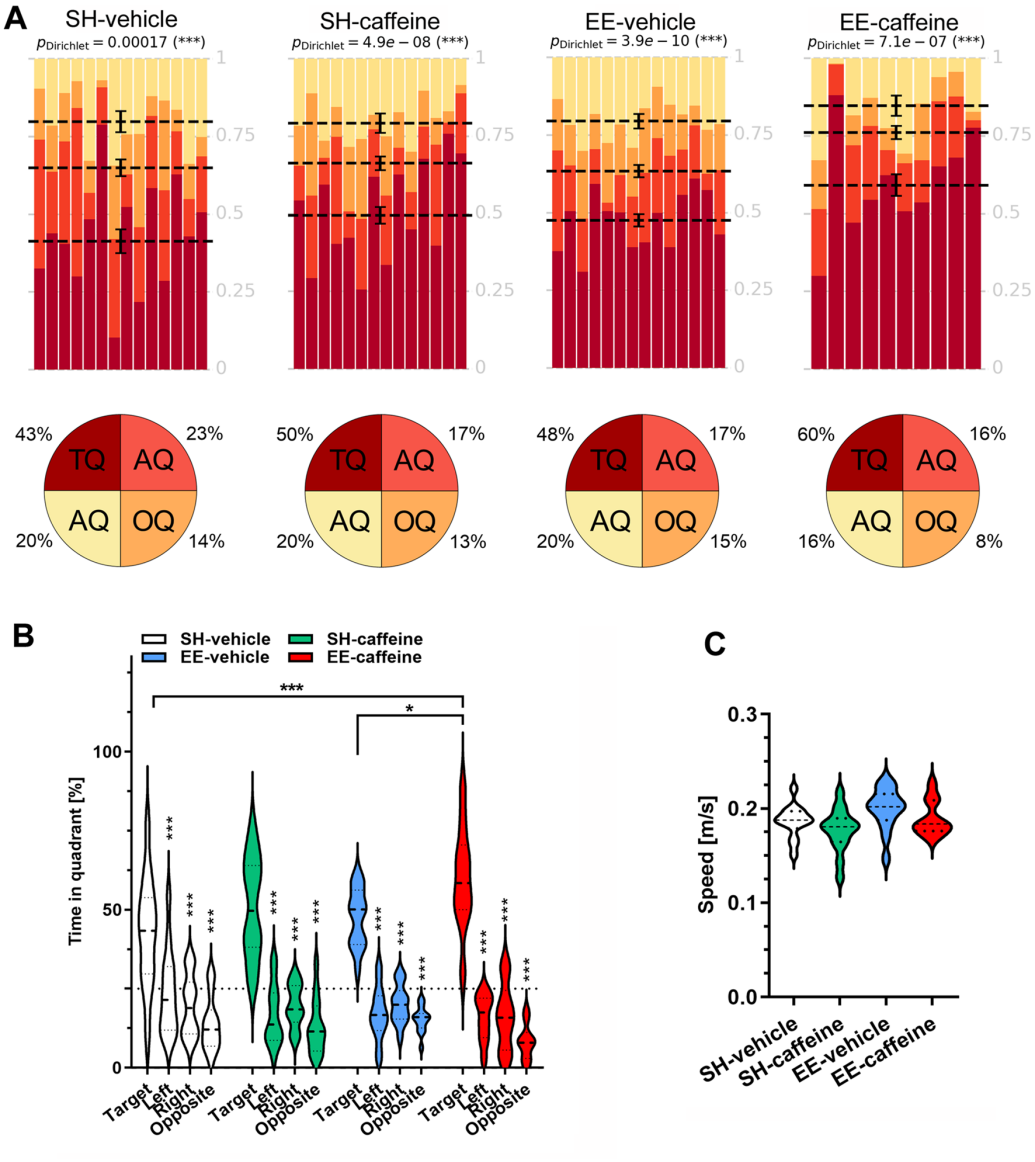
Improved spatial reference memory performance in WT mice upon EE housing and caffeine treatment. **A** All the groups displayed an intact spatial reference memory as they showed a clear and significant preference for the target compared to all the other quadrants. **B** Caffeine-treated, EE-housed mice spent significantly more time in the target quadrant when compared to vehicle-treated SH and EE mice. **C** Swimming speed during the probe trial showed no differences among the groups. Statistical analysis was performed using Dirichlet distributions (**A**) as described earlier [48]. One-way (**C**) and two-way ANOVA (**B**) followed by Bonferroni’s multiple comparisons test. *p < 0.05, ***p < 0.001. All data were given as mean ± SD

The day following the acquisition training, all mice completed the MWM probe trial. All groups showed a significant preference for the target quadrant (Fig. 4A), indicating that all the mice had learned the task. Notably, caffeine-treated mice housed under EE conditions showed a significantly improved spatial memory based on the average time the animals spent in the target quadrant as compared to their counterparts housed under standard conditions (Fig. 4B; two-way ANOVA followed by Bonferroni’s multiple comparison test; EE-caffeine vs. SH-vehicle, *p* < 0.001; EE-caffeine vs EE-vehicle, *p* < 0.05). However, the swimming speed did not differ (Fig. 4C). Likewise, no differences were observed in the overall number of target quadrant entries (Suppl. Fig. 2A), the latency to the first entry into the target quadrant (Suppl. Fig. 2B) or platform zone (Suppl. Fig. 2C), the time spent in the former platform position (Suppl. Fig. 2D), and the number of platform crossings (Suppl. Fig. 2E).

### CA1 neuron numbers and neurogenesis

**Fig. 5:**
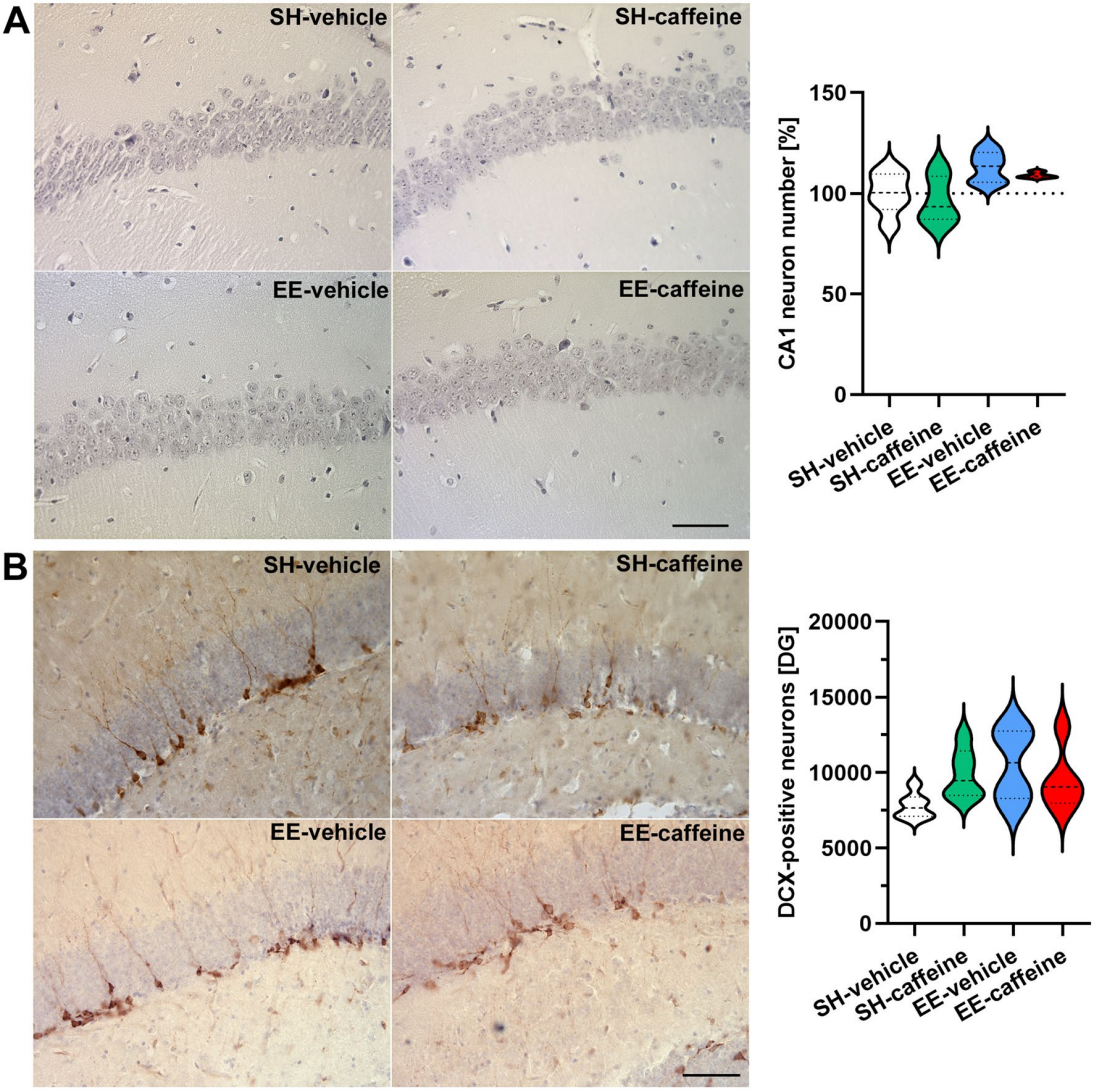
The effect of prolonged enrichment and caffeine treatment on hippocampal neuron numbers and neurogenesis in WT mice. **A** Exemplary images of haematoxylin-stained CA1 layers and quantification of CA1 neuron numbers. **B** Exemplary images of DCX-staining in the dentate gyrus and quantification of newborn DCX-positive neurons revealed no significant differences among the experimental groups. All data were given as mean ± SD

To evaluate whether the improved spatial reference memory and recognition memory performance can be related to an altered CA1 hippocampal neuron number or neurogenesis rate, CA1 neuron numbers and DCX-positive neurons were counted (Fig. 5). While the two groups housed under EE conditions showed higher CA1 neuron number compared to standard-housed WT mice, this difference barely reached statistical significance (one-way ANOVA, *p* = 0.052) (Fig. 5A). Neither the number of newborn neurons in the subgranular zone of the DG, represented by positive DCX-staining (one-way ANOVA, *p* = 0.068, Fig. 5B) nor the DG volume (one-way ANOVA, *p* = 0.429, Suppl. Fig. 3) was significantly altered between the different treatment groups.

### Increased hippocampal BDNF expression levels in mice housed under enriched environment conditions

**Fig. 6:**
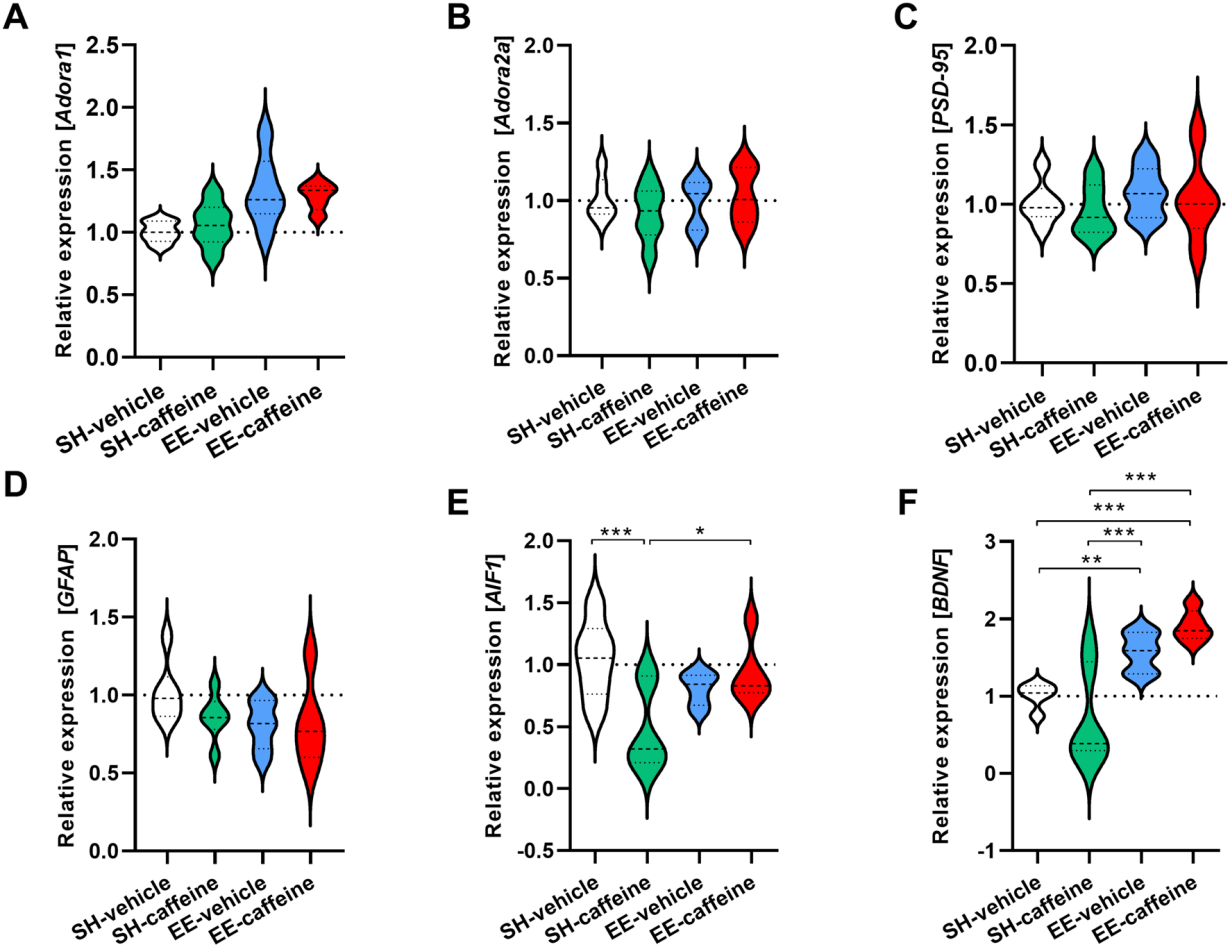
Hippocampal gene expression changes following long-term caffeine supplementation and enriched environment in C57Bl/6 mice. All four groups tested showed comparable expression of *Adora1* (**A**), *Adora2* (**B**), *PSD-95* (**C**) and *GFAP* (**D**) genes, whereas expression of *AIF1* was reduced in SH mice upon caffeine treatment (**E**). *BDNF* was significantly increased upon EE housing (**F**). One-way ANOVA was followed by Bonferroni’s multiple comparisons test. *p < 0.05, **p < 0.001, ***p < 0.001. All data were given as mean ± SD

To evaluate potential mechanisms for the observed synergistic effects of long-term caffeine treatment and exercise, expression levels of several candidate genes were analyzed in the hippocampus, with *β-Actin* serving as a housekeeping gene (Suppl. Fig. 4). Our results showed that caffeine, which is a known antagonist of adenosine receptors, did not alter the expression levels of the two adenosine receptors *Adora1* (Fig. 6A) and *Adora2* (Fig. 6B). Similarly, gene expression of the synaptic marker *PSD-95* (Fig. 6C) and the astrocytic marker *GFAP* (Fig. 6D) were unchanged. In contrast, the microglia marker *AIF1* (Iba1) showed a significant down-regulation in caffeine-treated compared to untreated mice housed under standard conditions, as well as to the EE-caffeine group (*p* < 0.001 and *p* < 0.05 respectively; Fig. 6E). A reduced number of hippocampal Iba1-positive microglia was also detected on the protein level with immunohistochemistry, which, however, did not reach statistical significance (Suppl. Fig.  5). Hippocampal gene expression levels of brain-derived neurotrophic factor (*BDNF*) were significantly increased in vehicle- and caffeine-treated EE-housed mice compared to their standard-housed control groups (Fig. 6F).

## Discussion

A variety of previous studies in rodents reported on beneficial effects of environmental enrichment or chronic caffeine consumption with regard to behavioural improvement. In the current study, we assessed whether a combination of increased physical activity within an enriched environment paradigm and chronic caffeine supplementation in drinking water may exert additive effects in terms of learning and memory as well as neuropathological alterations in C57BL/6J WT mice, which have been demonstrated to show age-related behavioural changes [61]. The enrichment paradigm, with or without caffeine supplementation, was started at the age of two months and was continued until the age of 6 months. A caffeine concentration of 300 mg/L has been used, resulting in considerable brain and plasma levels and corresponding to a daily intake of ~ 5 cups of coffee per day in humans [3, 4, 43]. The average daily water intake and body weight were assessed during the behavioural testing phase and no differences between caffeine- or vehicle-treated groups were detected. It has been previously shown that acute or chronic caffeine administration has anxiogenic-like effects in WT mice, resulting in reduced time periods spent in the open arms of the EPM [1, 71]. This was not observed in caffeine-treated mice housed under standard conditions. However, analysis of anxiety using the EPM paradigm revealed increased anxiety levels in caffeine-treated EE mice compared to the other groups, reflected by significantly reduced time spent in the open arms of the maze.

Long-term spatial reference memory was assessed using the well-established Morris water maze paradigm [54]. This task has been widely used to monitor learning and memory performance following enrichment in mouse models of AD [18, 32, 34, 70], as well as during normal aging [19, 33]. Though we used a different MWM protocol, our result of 43% target quadrant occupancy in SH-vehicle mice fits well with recently published data from a study analysing age-related cognitive decline in spatial learning and memory in C57BL/6J mice [27]. Chronic caffeine treatment has been shown to reverse memory deficits in this task in AD transgenic mice [4, 25, 63], and we did not find differences in this task in WT mice upon caffeine treatment in a recent study [63]. Analysis of target quadrant occupancy in the probe trial in the current study indicated that all groups learned the task, reflected by a significantly higher percentage of time spent in the target compared to all other quadrants. Remarkably, a comparison of the duration of stay in the target quadrant during the probe trial revealed a significantly increased occupancy in caffeine-treated EE-housed mice compared to SH-housed mice receiving tap water, indicating an additive effect of housing and caffeine supplementation. While we have shown previously that EE treatment increases target quadrant preference after 11 months of enrichment in C57Bl/6J mice [33], the current data may indicate that a shorter duration of the enrichment period might be not sufficient to see such an effect with this paradigm or alternatively, that such a phenotype becomes only visible upon age-dependent learning and memory decline, as published recently [27].

In recent years, several studies described the beneficial effects of EE housing on object recognition memory in rodent models of accelerated aging or AD [17, 24, 32, 56, 64]. Likewise, object recognition memory has also been reported to be improved upon physical exercise or EE housing in various strains of WT mice [38, 50, 52, 66]. Additionally, long-term caffeine treatment prevented the age-dependent deterioration in the object recognition task in 18-month-old CF1 mice, resulting in an outcome indistinguishable from 6-month-old control mice [11]. Housing in EE condition alone did not cause a significantly improved object recognition task performance in the present study, whereas the combination of enriched housing and caffeine supplementation resulted in a significantly higher discrimination index compared to the other experimental conditions. This underscores the hypothesis that caffeine supplementation together with increased physical activity gives rise to additive beneficial effects with regard to cognition.

In healthy adults, caffeine enhances exercise and cognitive performance in studies with measures of acute or endurance exercise [9, 23, 53, 62], and it is known that several parameters such as time of ingestion, dose or training status influence the efficacy of caffeine supplementation [7, 49]. Intake of caffeine in a performance bar can significantly improve endurance performance and complex cognitive abilities during and after exercise [29], providing some information on a combinatorial effect in the human situation. It has been shown previously that caffeine is able to trigger the secretion of BDNF from cultured hippocampal neurons [59], as well as in hippocampal slice cultures [42], albeit also decreased BDNF levels in the hippocampus of aged mice have been reported [11]. While we did not observe changes in *BDNF* gene expression levels upon caffeine consumption in standard-housed mice, our result of significantly increased *BDNF* levels upon EE housing supports previous studies [40, 72] and may at least partially explain the observed beneficial effects in learning and memory tasks. Additionally, the observed decreased *AIF1* expression in the hippocampus of caffeine-treated standard-housed mice in our study supports previous reports indicating that caffeine treatment attenuates neuroinflammation and microglia activation in rodent brain [6, 67].

With regard to neurogenesis, an inhibitory effect of 4-week caffeine consumption on the number of hippocampal neural precursors and learning and memory [26], as well as a depressed proliferation after 7-day administration of moderate to high doses has been described, with supraphysiological doses increasing the proliferation of neuronal precursors [68]. In contrast, in vitro data obtained from a human hippocampal progenitor cell line showed reduced progenitor integrity upon treatment with supraphysiological caffeine concentrations of 1.0 mM [30]. This is in contrast to our finding of unchanged neurogenesis rates in caffeine-treated SH- and EE-housed mice and might be attributed to long-term effects in the current study. On the other hand, these data support our observation of unchanged numbers of DCX-positive cells in a previous study employing a longer (11 months) enrichment protocol [33], though this might have also been influenced by the strongly decreased overall neurogenesis rates in aged rodents [41, 51].

In summary, we provide evidence for an additive beneficial effect of caffeine supplementation in combination with increased physical activity in learning and memory tasks in adult mice. Further studies are needed to evaluate whether such an approach may be an option to more efficiently ameliorate age-dependent cognitive decline and the development of neurodegenerative disorders such as AD.

## Supplementary Information

Below is the link to the electronic supplementary material.

Supplementary file1 (PDF 600 KB)
