# Supplementary material for: Combined long-term enriched environment and caffeine supplementation improve memory function in C57Bl6 mice
Source: Eur Arch Psychiatry Clin Neurosci. 2022 Jun 9;273(1):269–81. doi: 10.1007/s00406-022-01431-7 (PMC9958139; doi:10.1007/s00406-022-01431-7)
Supplement: Supplementary file 1 — Supplementary file1 (PDF 600 KB) [file 406_2022_1431_MOESM1_ESM.pdf]

Supplementary Information

Combined long-term enriched environment and caffeine supplementation improve memory function in C57Bl6 mice

M. Stazi, S. Zampar, M. Nadolny, L. Büschgens, T. Meyer, O. Wirths

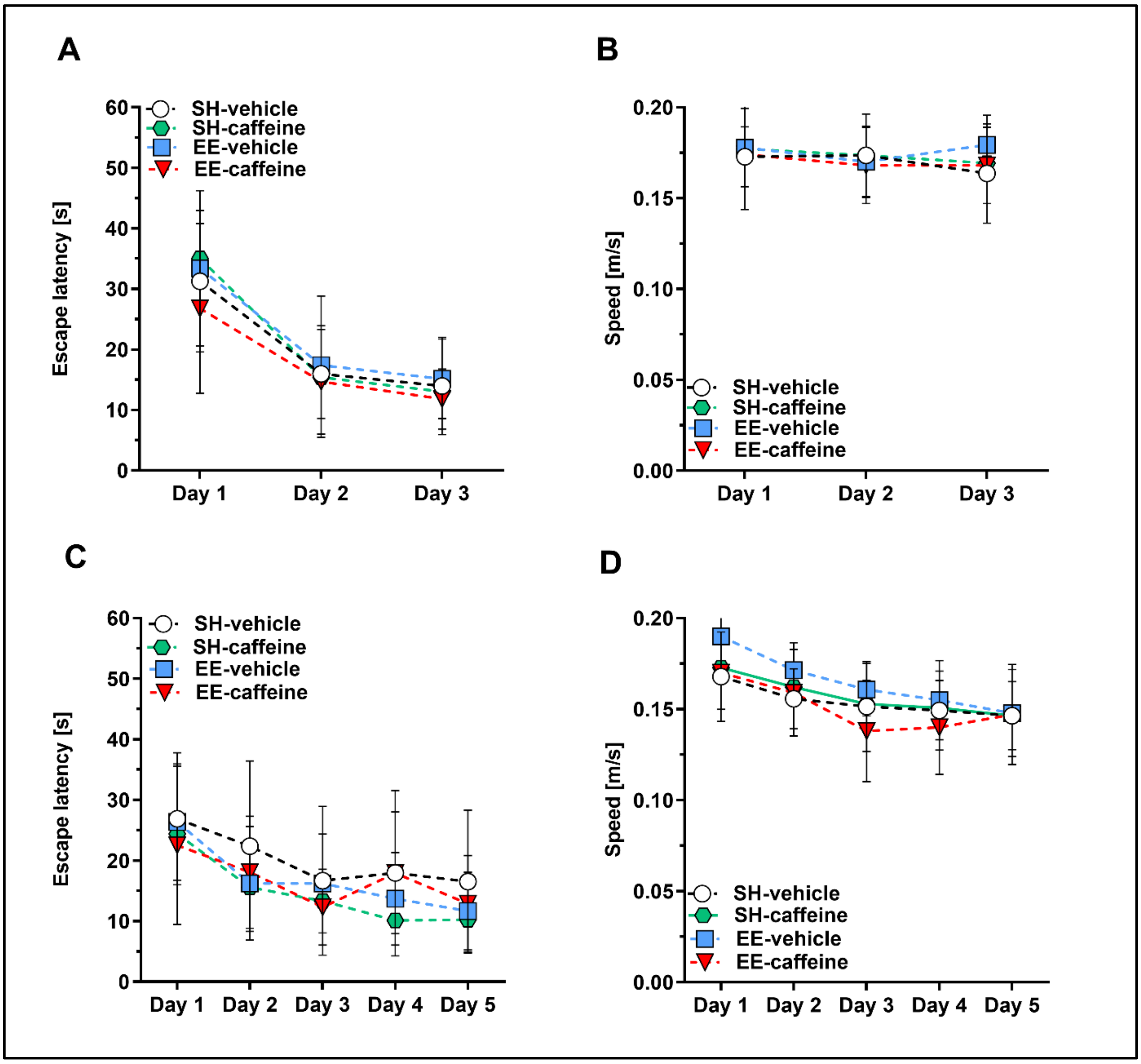

Supplementary Figure 1: All groups showed progressively decreasing escape latencies over the 3 days of cued training (A) and over the 5 days of acquisition training (C). During the cued and acquisition phase, no differences in swimming speed were observed among the different experimental groups (B, D).

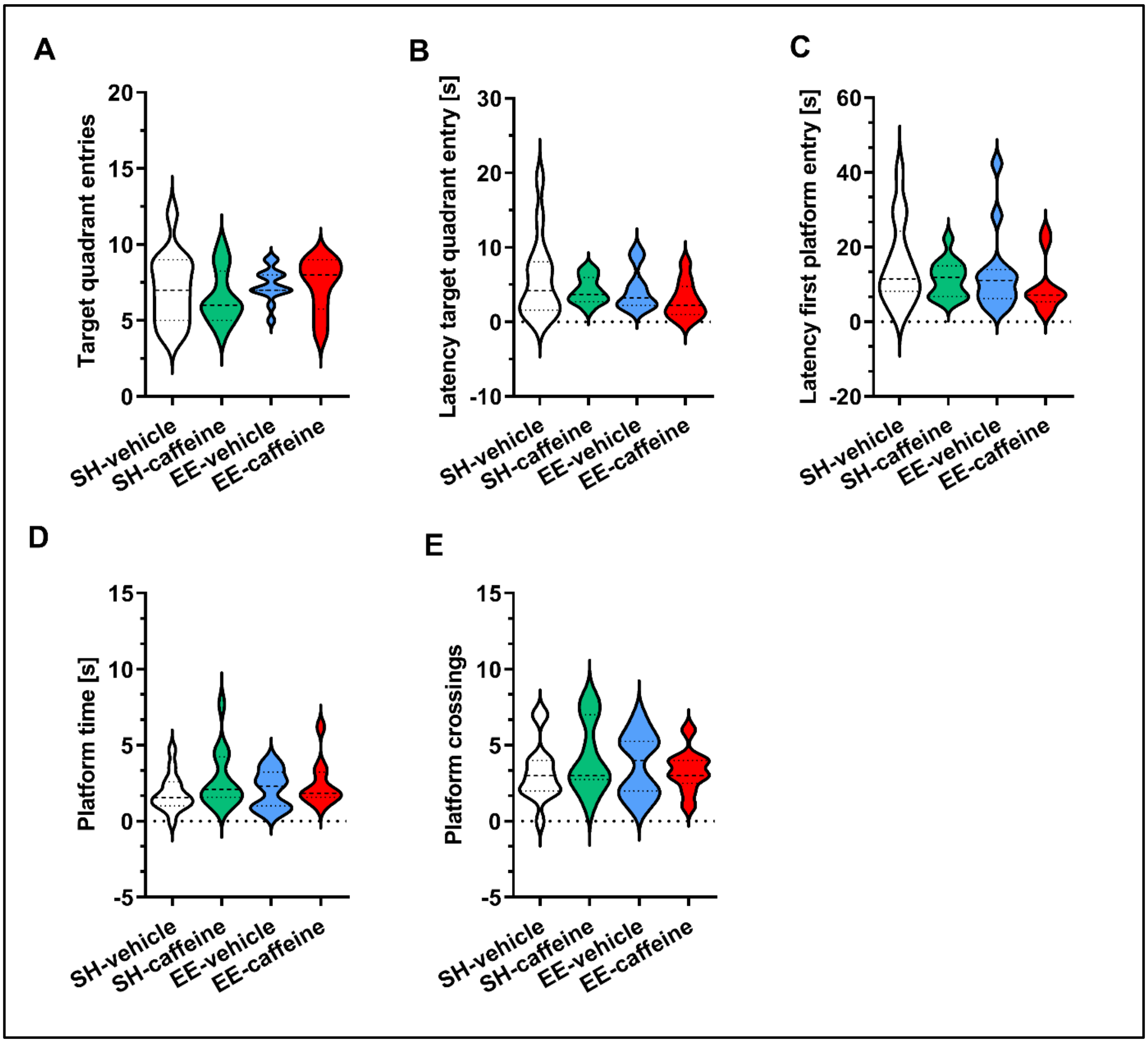

Supplementary Figure 2: No differences were observed in the overall number of target quadrant entries (A), the latency to the first entry into the target quadrant (B) or platform zone (C), the time spent in the former platform position (D) or the number of platform crossings (E).

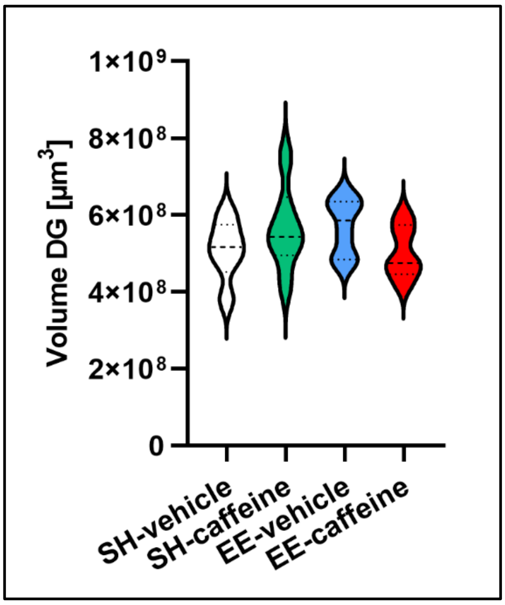

Supplementary Figure 3: No differences in dentate gyrus volume were detected among the different experimental groups.

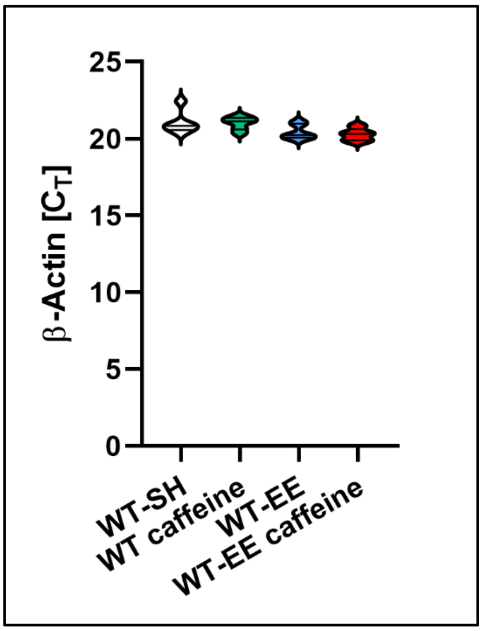

Supplementary Figure 4: Unaltered threshold cycle (C_T) values for the housekeeping gene β-Actin in the RT-PCR analysis among the different experimental groups.

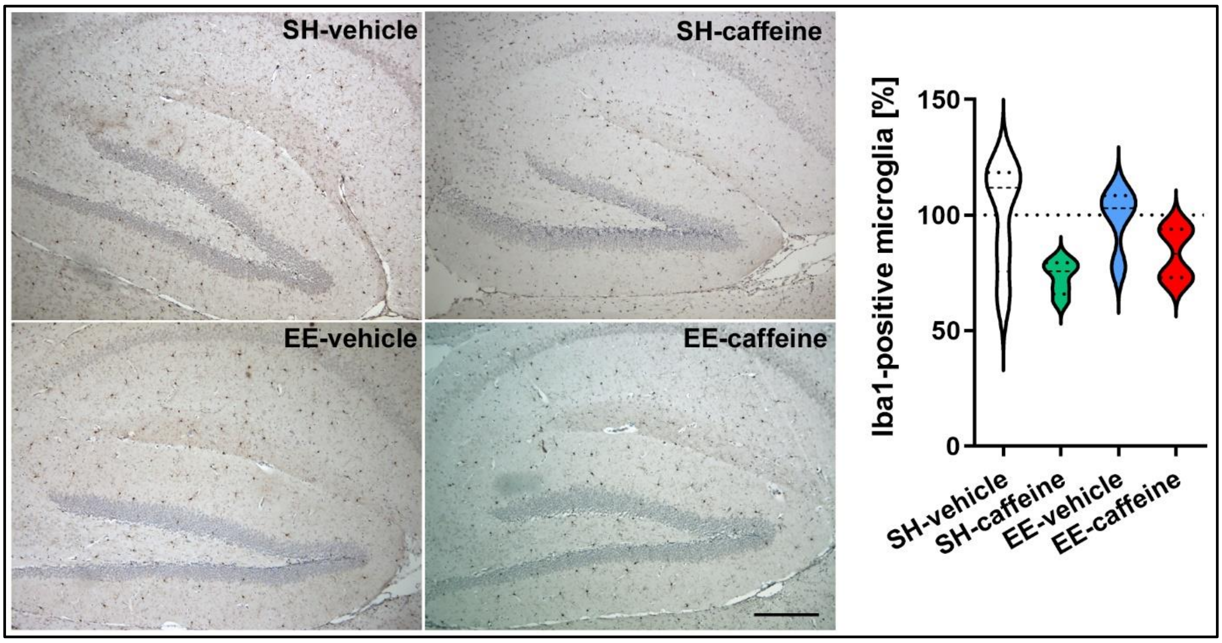

Supplementary Figure 5: Quantification of Iba1-positive microglial cells in the hippocampus revealed a reduced number of microglia in standard-housed caffeine-treated mice, which however, did not reach statistical significance (*p*ANOVA = 0.0624).

Supplementary Material and Methods

Immunohistochemistry of Iba1-positive microglia

Sagittal paraffin-embedded brain samples (n = 4-5 per group) were cut at 4 µm thickness. Paraffin was removed by incubating the slides in xylol and sections were rehydrated with an ascending ethanol series. Endogenous peroxidases were blocked with a 30 min treatment of 0.3% H_2O_2 in 0.01 M phosphate-buffered saline (PBS) and antigens were retrieved by boiling sections in 0.01 M citrate buffer (pH 6.0). An incubation step in 0.01 M PBS incl. 4% skim milk and 10% fetal cow serum was applied for 1 h to block unspecific binding sites. A guinea pig anti-Iba1 antibody (1:500; #234004, Synaptic Systems) was applied overnight at room temperature in a humid chamber. Biotinylated secondary antibodies (1:200, Dianova) were applied for 1 h and staining was visualized via the ABC method using the Vectastain kit (Vector Laboratories, Burlingame, CA, USA) with diaminobenzidine (DAB) as a chromogen and haematoxylin counterstaining.

Images of the CA1 region of the hippocampus from DAB-stained sections were taken (n = 4 sections per animal), using an Olympus BX-51 microscope equipped with a Moticam pro 282 camera (Motic, Wetzlar, Germany. The captured images were analysed using the point-counting tool implemented in the ImageJ software package (https://imagej.nih.gov/ij/).
